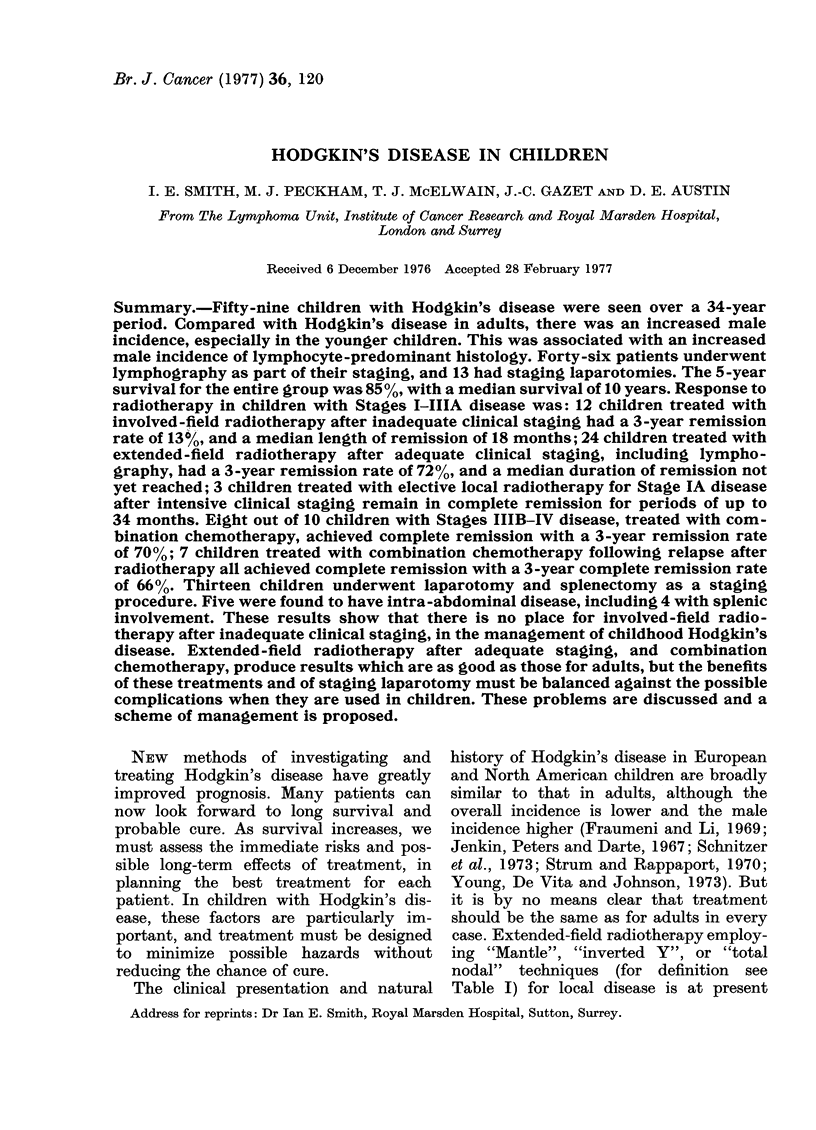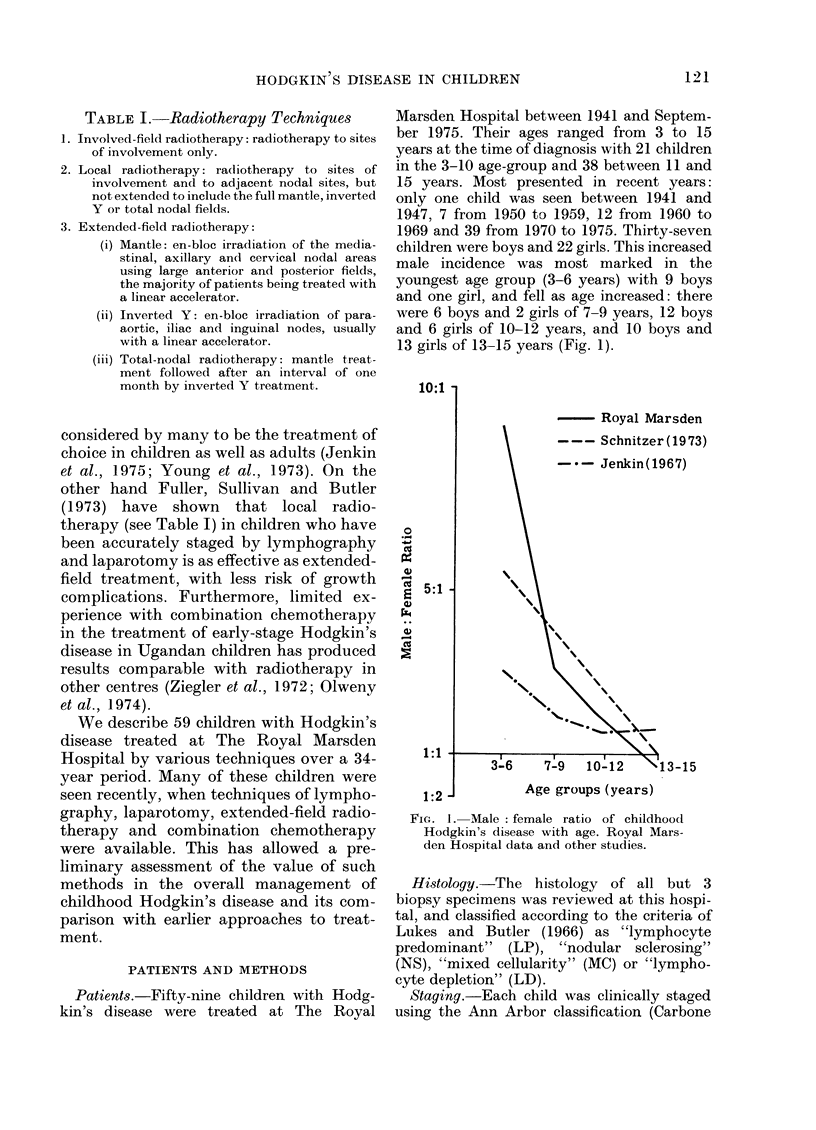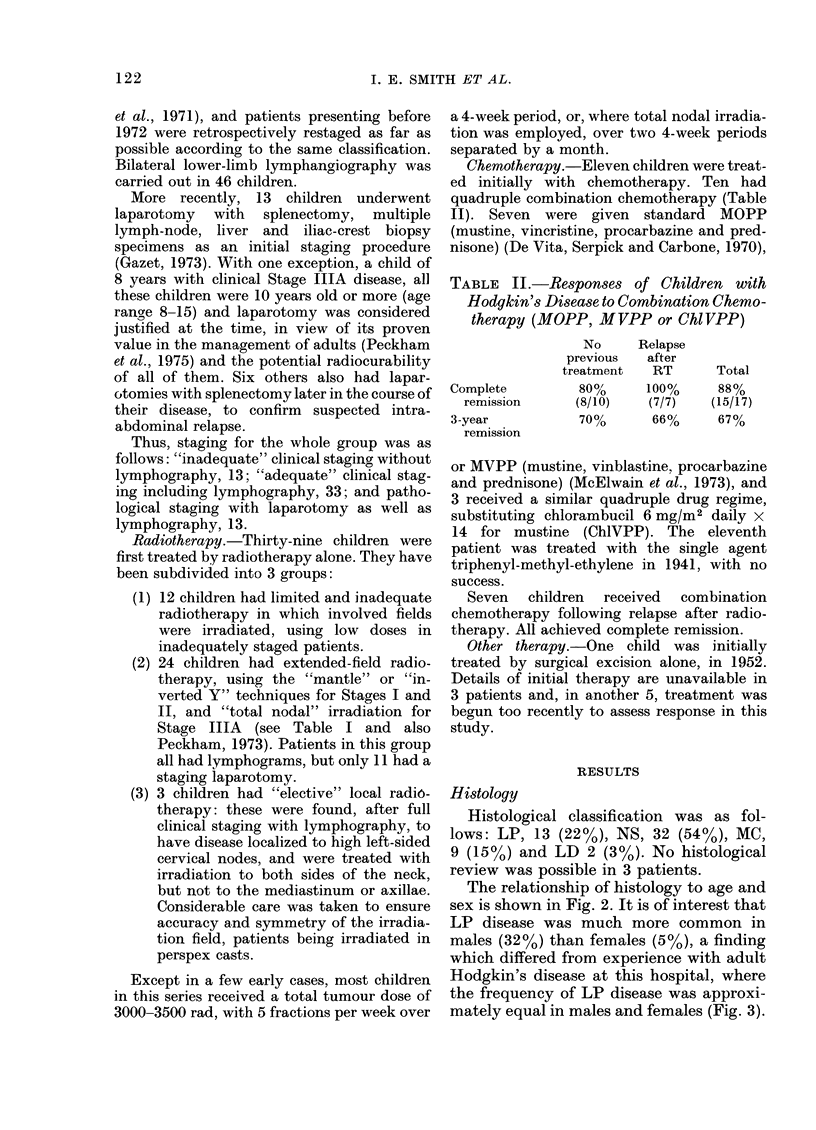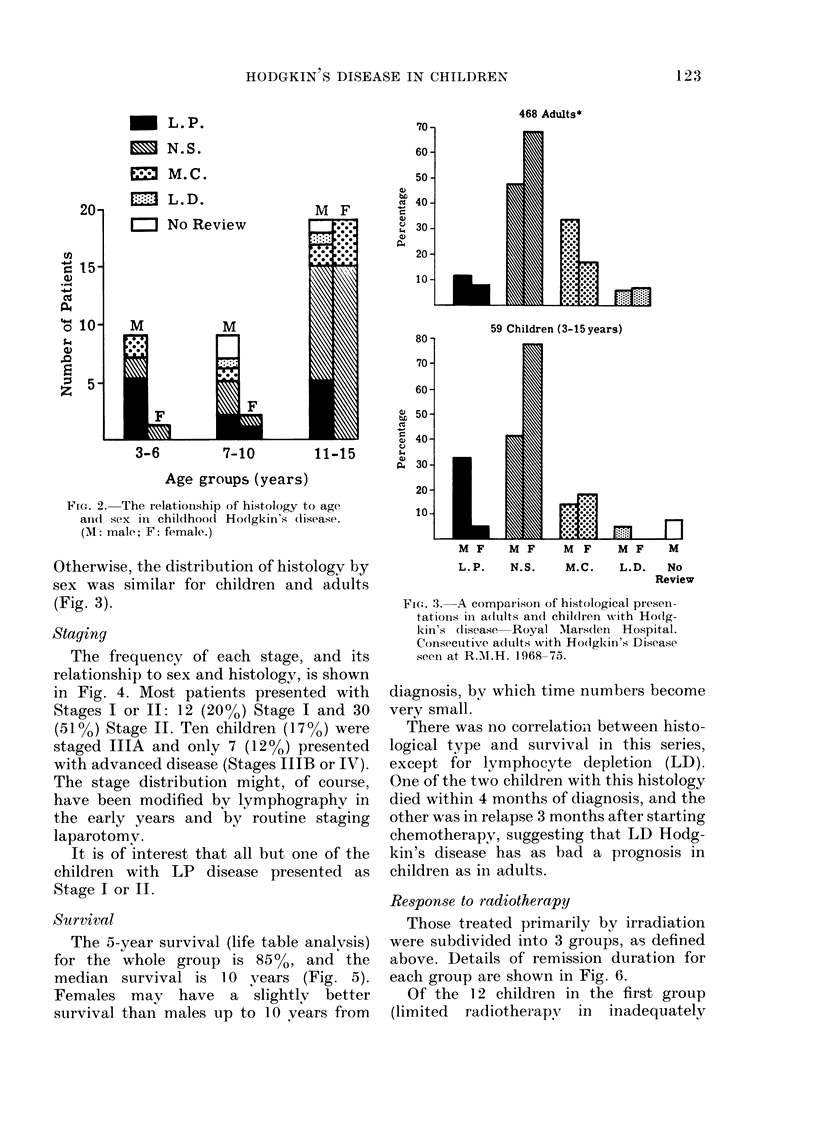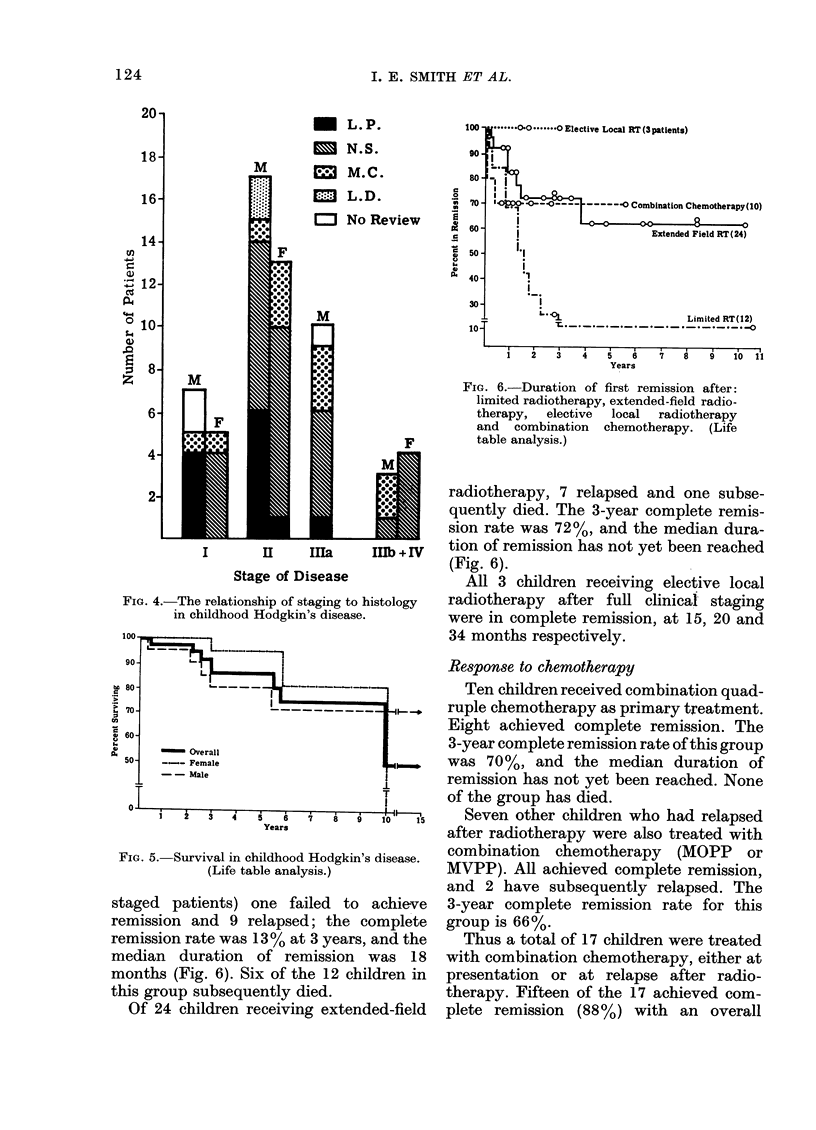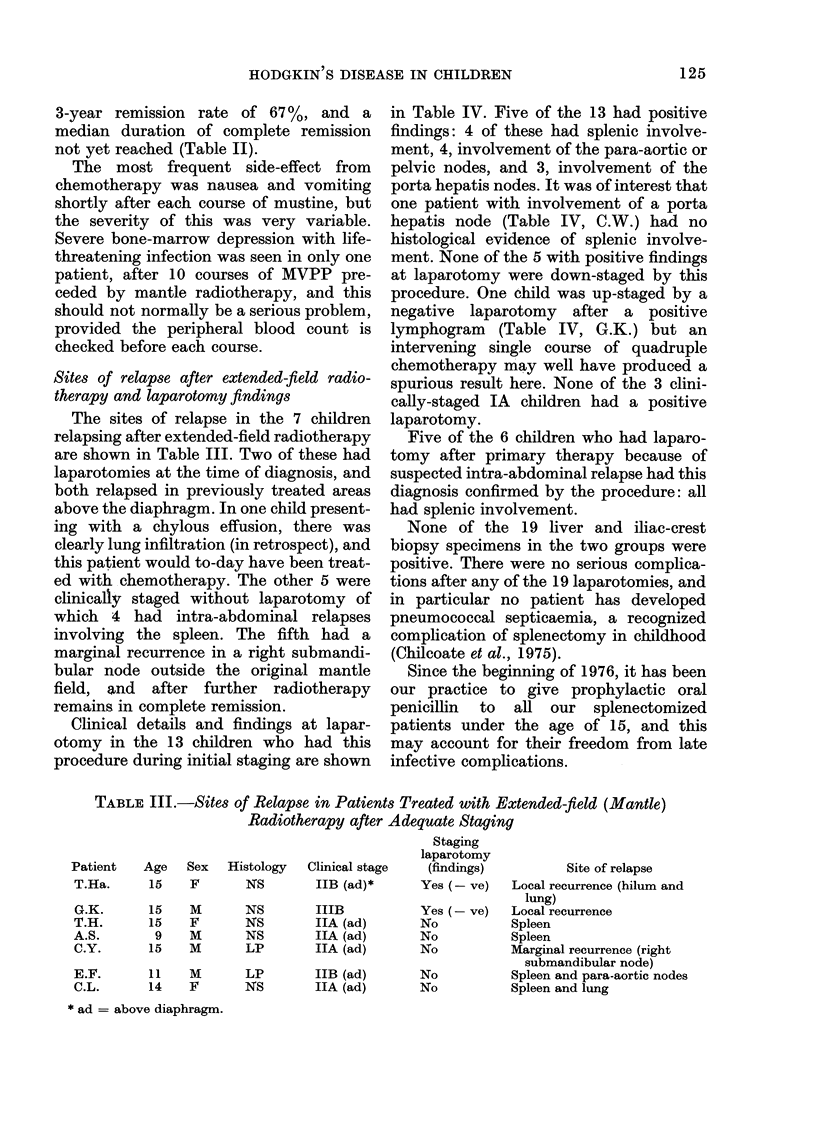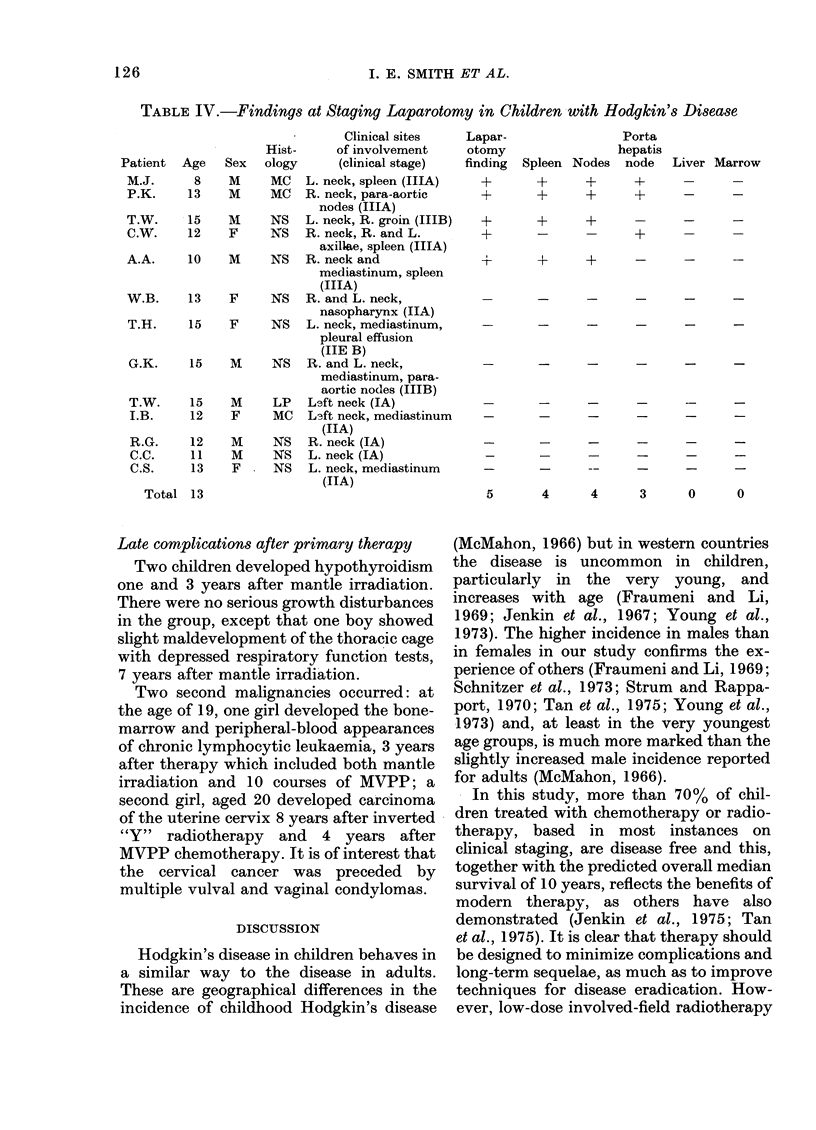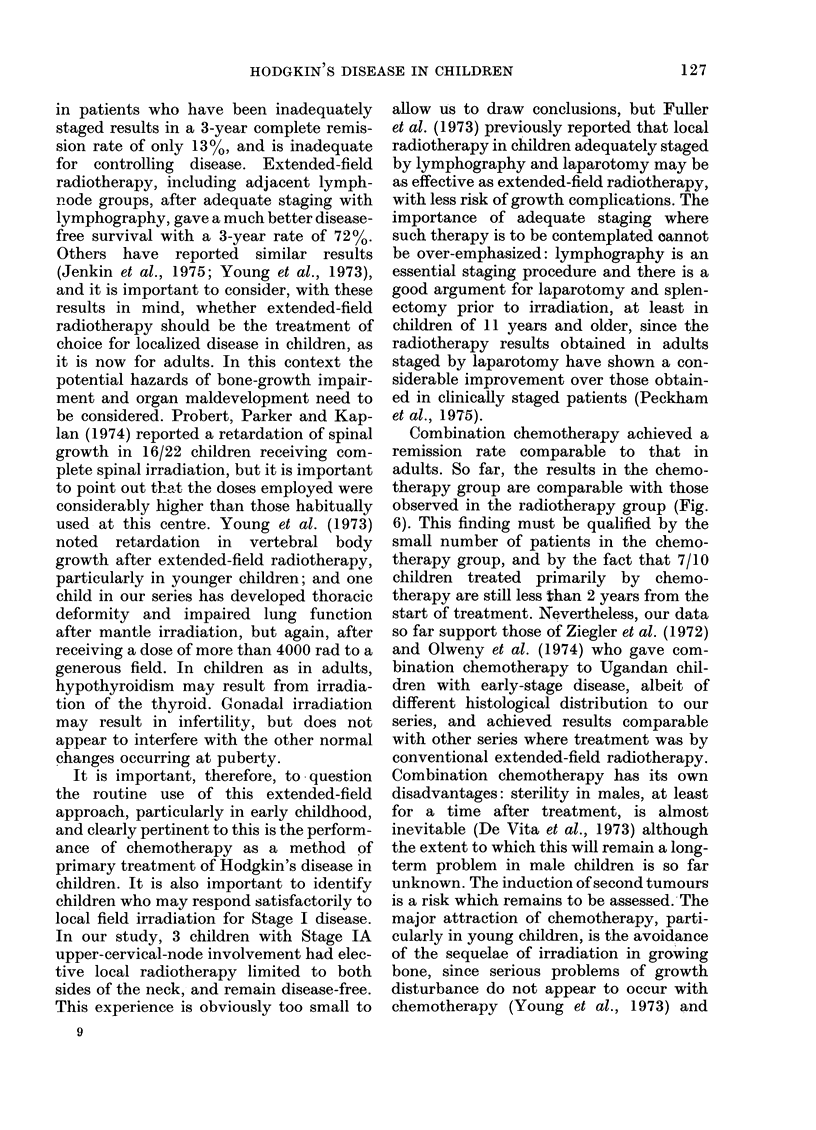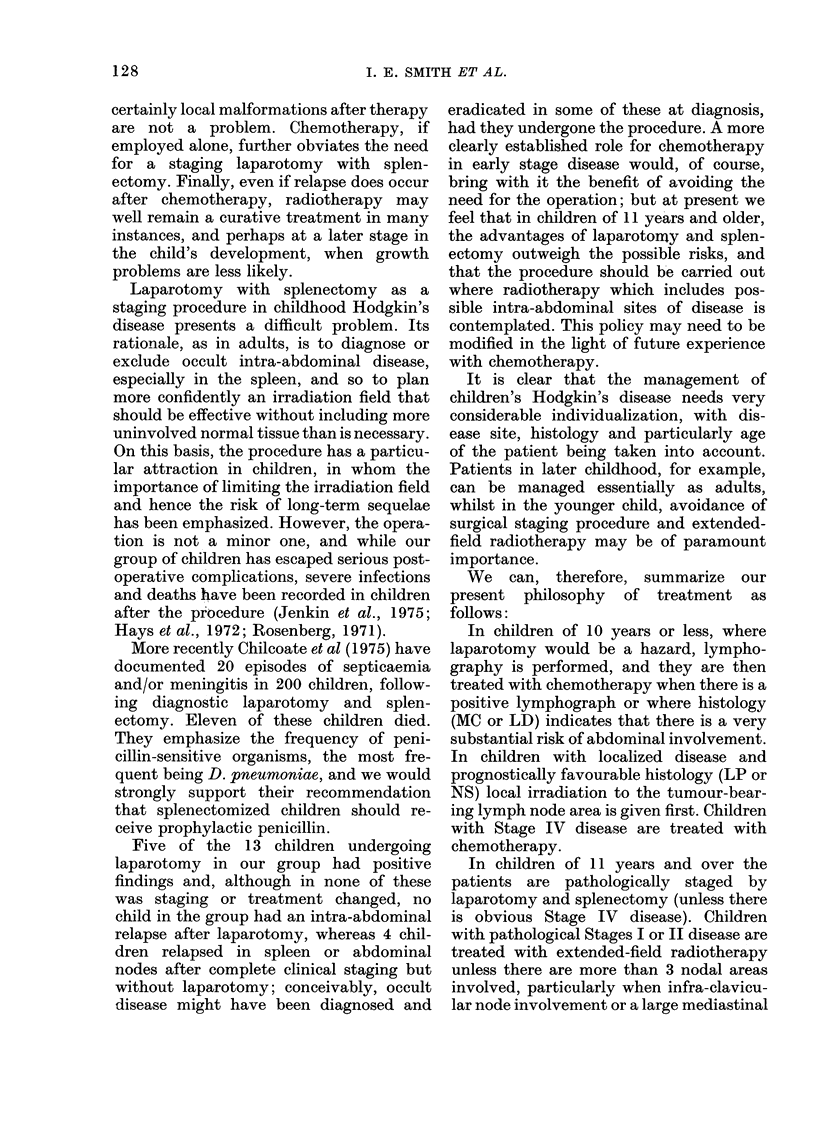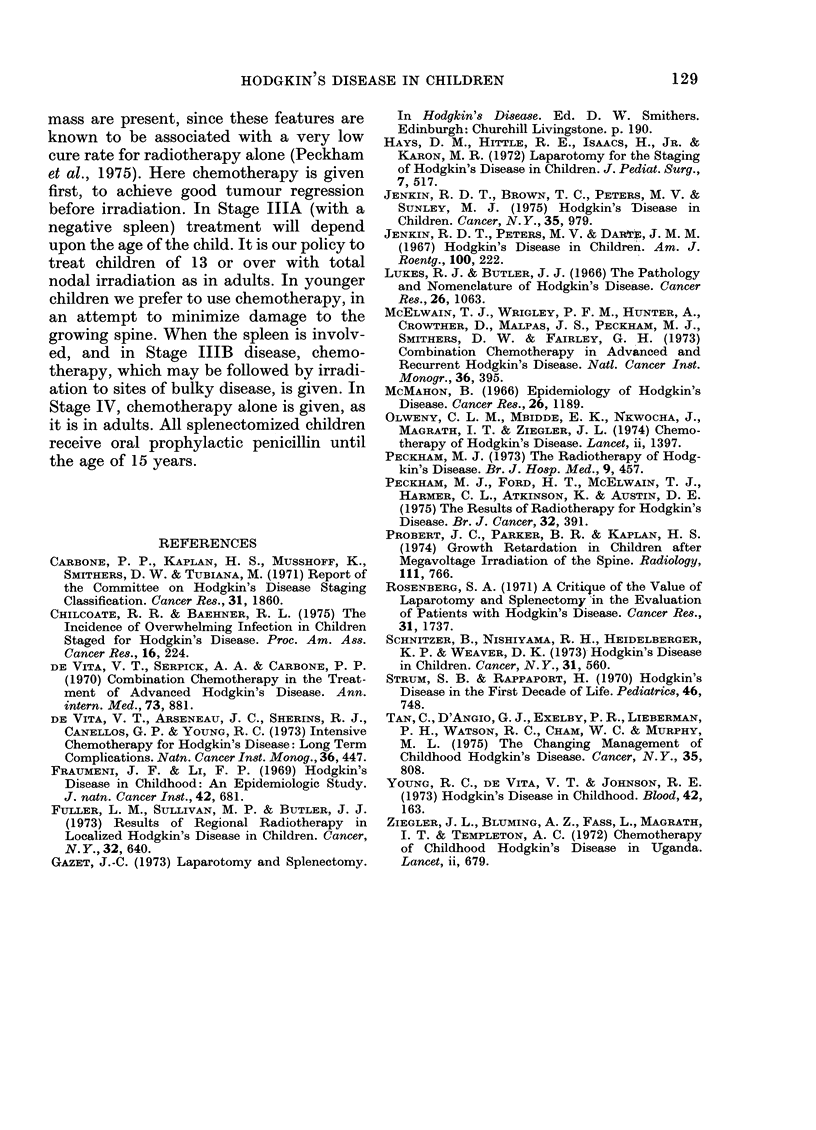# Hodgkin's Disease in Children

**DOI:** 10.1038/bjc.1977.162

**Published:** 1977-07

**Authors:** I. E. Smith, M. J. Peckham, T. J. McElwain, J.-C. Gazet, D. E. Austin

## Abstract

Fifty-nine children with Hodgkin's disease were seen over a 34-year period. Compared with Hodgkin's disease in adults, there was an increased male incidence, especially in the younger children. This was associated with an increased male incidence of lymphocyte-predominant histology. Forty-six patients underwent lymphography as part of their staging, and 13 had staging laparotomies. The 5-year survival for the entire group was 85%, with a median survival of 10 years. Response to radiotherapy in children with Stages I-IIIA disease was: 12 children treated with involved-field radiotherapy after inadequate clinical staging had a 3-year remission rate of 13%, and a median length of remission of 18 months; 24 children treated with extended-field radiotherapy after adequate clinical staging, including lymphography, had a 3-year remission rate of 72%, and a median duration of remission not yet reached; 3 children treated with elective local radiotherapy for Stage IA disease after intensive clinical staging remain in complete remission for periods of up to 34 months. Eight out of 10 children with Stages IIIB-IV disease, treated with combination chemotherapy, achieved complete remission with a 3-year remission rate of 70%; 7 children treated with combination chemotherapy following relapse after radiotherapy all achieved complete remission with a 3-year complete remission rate of 66%. Thirteen children underwent laparotomy and splenectomy as a staging procedure. Five were found to have intra-abdominal disease, including 4 with splenic involvement. These results show that there is no place for involved-field radiotherapy after inadequate clinical staging, in the management of childhood Hodgkin's disease. Extended-field radiotherapy after adequate staging, and combination chemotherapy, produce results which are as good as those for adults, but the benefits of these treatments and of staging laparotomy must be balanced against the possible complications when they are used in children. These problems are discussed and a scheme of management is proposed.


*Br. J. Cancer* (1977) **36**, 120

### HODGKIN'S DISEASE IN CHILDREN

I. E. SMITH, M. J. PECKHAM, T. J. McELWAIN, J.-C. GAZET AND D. E. AUSTIN

*From The Lymphoma Unit, Institute of Cancer Research and Royal Marsden Hospital, London and Surrey*

Received 6 December 1976 Accepted 28 February 1977

**Summary.—Fifty-nine children with Hodgkin's disease were seen over a 34-year period. Compared with Hodgkin's disease in adults, there was an increased male incidence, especially in the younger children. This was associated with an increased male incidence of lymphocyte-predominant histology. Forty-six patients underwent lymphography as part of their staging, and 13 had staging laparotomies. The 5-year survival for the entire group was 85%, with a median survival of 10 years. Response to radiotherapy in children with Stages I–IIIA disease was: 12 children treated with involved-field radiotherapy after inadequate clinical staging had a 3-year remission rate of 13%, and a median length of remission of 18 months; 24 children treated with extended-field radiotherapy after adequate clinical staging, including lymphography, had a 3-year remission rate of 72%, and a median duration of remission not yet reached; 3 children treated with elective local radiotherapy for Stage IA disease after intensive clinical staging remain in complete remission for periods of up to 34 months. Eight out of 10 children with Stages IIIB–IV disease, treated with combination chemotherapy, achieved complete remission with a 3-year remission rate of 70%; 7 children treated with combination chemotherapy following relapse after radiotherapy all achieved complete remission with a 3-year complete remission rate of 66%. Thirteen children underwent laparotomy and splenectomy as a staging procedure. Five were found to have intra-abdominal disease, including 4 with splenic involvement. These results show that there is no place for involved-field radiotherapy after inadequate clinical staging, in the management of childhood Hodgkin's disease. Extended-field radiotherapy after adequate staging, and combination chemotherapy, produce results which are as good as those for adults, but the benefits of these treatments and of staging laparotomy must be balanced against the possible complications when they are used in children. These problems are discussed and a scheme of management is proposed.**

NEW methods of investigating and treating Hodgkin's disease have greatly improved prognosis. Many patients can now look forward to long survival and probable cure. As survival increases, we must assess the immediate risks and possible long-term effects of treatment, in planning the best treatment for each patient. In children with Hodgkin's disease, these factors are particularly important, and treatment must be designed to minimize possible hazards without reducing the chance of cure.

The clinical presentation and natural history of Hodgkin's disease in European and North American children are broadly similar to that in adults, although the overall incidence is lower and the male incidence higher (Fraumeni and Li, 1969; Jenkin, Peters and Darte, 1967; Schnitzer *et al.*, 1973; Strum and Rappaport, 1970; Young, De Vita and Johnson, 1973). But it is by no means clear that treatment should be the same as for adults in every case. Extended-field radiotherapy employing "Mantle", "inverted Y", or "total nodal" techniques (for definition see Table I) for local disease is at present

Address for reprints: Dr Ian E. Smith, Royal Marsden Hospital, Sutton, Surrey.

TABLE I.—*Radiotherapy Techniques*

1. Involved-field radiotherapy: radiotherapy to sites of involvement only.
2. Local radiotherapy: radiotherapy to sites of involvement and to adjacent nodal sites, but not extended to include the full mantle, inverted Y or total nodal fields.
3. Extended-field radiotherapy:
   (i) Mantle: en-bloc irradiation of the mediastinal, axillary and cervical nodal areas using large anterior and posterior fields, the majority of patients being treated with a linear accelerator.
   (ii) Inverted Y: en-bloc irradiation of para-aortic, iliac and inguinal nodes, usually with a linear accelerator.
   (iii) Total-nodal radiotherapy: mantle treatment followed after an interval of one month by inverted Y treatment.

considered by many to be the treatment of choice in children as well as adults (Jenkin *et al.*, 1975; Young *et al.*, 1973). On the other hand Fuller, Sullivan and Butler (1973) have shown that local radiotherapy (see Table I) in children who have been accurately staged by lymphography and laparotomy is as effective as extended-field treatment, with less risk of growth complications. Furthermore, limited experience with combination chemotherapy in the treatment of early-stage Hodgkin's disease in Ugandan children has produced results comparable with radiotherapy in other centres (Ziegler *et al.*, 1972; Olweny *et al.*, 1974).

We describe 59 children with Hodgkin's disease treated at The Royal Marsden Hospital by various techniques over a 34-year period. Many of these children were seen recently, when techniques of lymphography, laparotomy, extended-field radiotherapy and combination chemotherapy were available. This has allowed a preliminary assessment of the value of such methods in the overall management of childhood Hodgkin's disease and its comparison with earlier approaches to treatment.

### PATIENTS AND METHODS

*Patients.*—Fifty-nine children with Hodgkin's disease were treated at The Royal Marsden Hospital between 1941 and September 1975. Their ages ranged from 3 to 15 years at the time of diagnosis with 21 children in the 3–10 age-group and 38 between 11 and 15 years. Most presented in recent years: only one child was seen between 1941 and 1947, 7 from 1950 to 1959, 12 from 1960 to 1969 and 39 from 1970 to 1975. Thirty-seven children were boys and 22 girls. This increased male incidence was most marked in the youngest age group (3–6 years) with 9 boys and one girl, and fell as age increased: there were 6 boys and 2 girls of 7–9 years, 12 boys and 6 girls of 10–12 years, and 10 boys and 13 girls of 13–15 years (Fig. 1).

FIG. 1.—Male : female ratio of childhood Hodgkin's disease with age. Royal Marsden Hospital data and other studies.

*Histology.*—The histology of all but 3 biopsy specimens was reviewed at this hospital, and classified according to the criteria of Lukes and Butler (1966) as "lymphocyte predominant" (LP), "nodular sclerosing" (NS), "mixed cellularity" (MC) or "lymphocyte depletion" (LD).

*Staging.*—Each child was clinically staged using the Ann Arbor classification (Carbone

*et al.*, 1971), and patients presenting before 1972 were retrospectively restaged as far as possible according to the same classification. Bilateral lower-limb lymphangiography was carried out in 46 children.

More recently, 13 children underwent laparotomy with splenectomy, multiple lymph-node, liver and iliac-crest biopsy specimens as an initial staging procedure (Gazet, 1973). With one exception, a child of 8 years with clinical Stage IIIA disease, all these children were 10 years old or more (age range 8–15) and laparotomy was considered justified at the time, in view of its proven value in the management of adults (Peckham *et al.*, 1975) and the potential radiocurability of all of them. Six others also had laparotomies with splenectomy later in the course of their disease, to confirm suspected intra-abdominal relapse.

Thus, staging for the whole group was as follows: "inadequate" clinical staging without lymphography, 13; "adequate" clinical staging including lymphography, 33; and pathological staging with laparotomy as well as lymphography, 13.

*Radiotherapy.*—Thirty-nine children were first treated by radiotherapy alone. They have been subdivided into 3 groups:

(1) 12 children had limited and inadequate radiotherapy in which involved fields were irradiated, using low doses in inadequately staged patients.
(2) 24 children had extended-field radiotherapy, using the "mantle" or "inverted Y" techniques for Stages I and II, and "total nodal" irradiation for Stage IIIA (see Table I and also Peckham, 1973). Patients in this group all had lymphograms, but only 11 had a staging laparotomy.
(3) 3 children had "elective" local radiotherapy: these were found, after full clinical staging with lymphography, to have disease localized to high left-sided cervical nodes, and were treated with irradiation to both sides of the neck, but not to the mediastinum or axillae. Considerable care was taken to ensure accuracy and symmetry of the irradiation field, patients being irradiated in perspex casts.

Except in a few early cases, most children in this series received a total tumour dose of 3000–3500 rad, with 5 fractions per week over a 4-week period, or, where total nodal irradiation was employed, over two 4-week periods separated by a month.

*Chemotherapy.*—Eleven children were treated initially with chemotherapy. Ten had quadruple combination chemotherapy (Table II). Seven were given standard MOPP (mustine, vincristine, procarbazine and prednisone) (De Vita, Serpick and Carbone, 1970),

TABLE II.—*Responses of Children with Hodgkin's Disease to Combination Chemotherapy (MOPP, MVPP or ChlVPP)*

| | No previous treatment | Relapse after RT | Total |
|---|---|---|---|
| Complete remission | 80% (8/10) | 100% (7/7) | 88% (15/17) |
| 3-year remission | 70% | 66% | 67% |

or MVPP (mustine, vinblastine, procarbazine and prednisone) (McElwain *et al.*, 1973), and 3 received a similar quadruple drug regime, substituting chlorambucil 6 mg/m$^2$ daily $\times$ 14 for mustine (ChlVPP). The eleventh patient was treated with the single agent triphenyl-methyl-ethylene in 1941, with no success.

Seven children received combination chemotherapy following relapse after radiotherapy. All achieved complete remission.

*Other therapy.*—One child was initially treated by surgical excision alone, in 1952. Details of initial therapy are unavailable in 3 patients and, in another 5, treatment was begun too recently to assess response in this study.

### RESULTS

### *Histology*

Histological classification was as follows: LP, 13 (22%), NS, 32 (54%), MC, 9 (15%) and LD 2 (3%). No histological review was possible in 3 patients.

The relationship of histology to age and sex is shown in Fig. 2. It is of interest that LP disease was much more common in males (32%) than females (5%), a finding which differed from experience with adult Hodgkin's disease at this hospital, where the frequency of LP disease was approximately equal in males and females (Fig. 3).

FIG. 2.—The relationship of histology to age and sex in childhood Hodgkin's disease. (M: male; F: female.)

FIG. 3.—A comparison of histological presentations in adults and children with Hodgkin's disease—Royal Marsden Hospital. Consecutive adults with Hodgkin's Disease seen at R.M.H. 1968–75.

Otherwise, the distribution of histology by sex was similar for children and adults (Fig. 3).

*Staging*

The frequency of each stage, and its relationship to sex and histology, is shown in Fig. 4. Most patients presented with Stages I or II: 12 (20%) Stage I and 30 (51%) Stage II. Ten children (17%) were staged IIIA and only 7 (12%) presented with advanced disease (Stages IIIB or IV). The stage distribution might, of course, have been modified by lymphography in the early years and by routine staging laparotomy.

It is of interest that all but one of the children with LP disease presented as Stage I or II.

*Survival*

The 5-year survival (life table analysis) for the whole group is 85%, and the median survival is 10 years (Fig. 5). Females may have a slightly better survival than males up to 10 years from diagnosis, by which time numbers become very small.

There was no correlation between histological type and survival in this series, except for lymphocyte depletion (LD). One of the two children with this histology died within 4 months of diagnosis, and the other was in relapse 3 months after starting chemotherapy, suggesting that LD Hodgkin's disease has as bad a prognosis in children as in adults.

*Response to radiotherapy*

Those treated primarily by irradiation were subdivided into 3 groups, as defined above. Details of remission duration for each group are shown in Fig. 6.

Of the 12 children in the first group (limited radiotherapy in inadequately

FIG. 4.—The relationship of staging to histology in childhood Hodgkin's disease.

FIG. 5.—Survival in childhood Hodgkin's disease. (Life table analysis.)

staged patients) one failed to achieve remission and 9 relapsed; the complete remission rate was 13% at 3 years, and the median duration of remission was 18 months (Fig. 6). Six of the 12 children in this group subsequently died.

Of 24 children receiving extended-field

FIG. 6.—Duration of first remission after: limited radiotherapy, extended-field radiotherapy, elective local radiotherapy and combination chemotherapy. (Life table analysis.)

radiotherapy, 7 relapsed and one subsequently died. The 3-year complete remission rate was 72%, and the median duration of remission has not yet been reached (Fig. 6).

All 3 children receiving elective local radiotherapy after full clinical staging were in complete remission, at 15, 20 and 34 months respectively.

*Response to chemotherapy*

Ten children received combination quadruple chemotherapy as primary treatment. Eight achieved complete remission. The 3-year complete remission rate of this group was 70%, and the median duration of remission has not yet been reached. None of the group has died.

Seven other children who had relapsed after radiotherapy were also treated with combination chemotherapy (MOPP or MVPP). All achieved complete remission, and 2 have subsequently relapsed. The 3-year complete remission rate for this group is 66%.

Thus a total of 17 children were treated with combination chemotherapy, either at presentation or at relapse after radiotherapy. Fifteen of the 17 achieved complete remission (88%) with an overall

3-year remission rate of 67%, and a median duration of complete remission not yet reached (Table II).

The most frequent side-effect from chemotherapy was nausea and vomiting shortly after each course of mustine, but the severity of this was very variable. Severe bone-marrow depression with life-threatening infection was seen in only one patient, after 10 courses of MVPP preceded by mantle radiotherapy, and this should not normally be a serious problem, provided the peripheral blood count is checked before each course.

*Sites of relapse after extended-field radiotherapy and laparotomy findings*

The sites of relapse in the 7 children relapsing after extended-field radiotherapy are shown in Table III. Two of these had laparotomies at the time of diagnosis, and both relapsed in previously treated areas above the diaphragm. In one child presenting with a chylous effusion, there was clearly lung infiltration (in retrospect), and this patient would to-day have been treated with chemotherapy. The other 5 were clinically staged without laparotomy of which 4 had intra-abdominal relapses involving the spleen. The fifth had a marginal recurrence in a right submandibular node outside the original mantle field, and after further radiotherapy remains in complete remission.

Clinical details and findings at laparotomy in the 13 children who had this procedure during initial staging are shown in Table IV. Five of the 13 had positive findings: 4 of these had splenic involvement, 4, involvement of the para-aortic or pelvic nodes, and 3, involvement of the porta hepatis nodes. It was of interest that one patient with involvement of a porta hepatis node (Table IV, C.W.) had no histological evidence of splenic involvement. None of the 5 with positive findings at laparotomy were down-staged by this procedure. One child was up-staged by a negative laparotomy after a positive lymphogram (Table IV, G.K.) but an intervening single course of quadruple chemotherapy may well have produced a spurious result here. None of the 3 clinically-staged IA children had a positive laparotomy.

Five of the 6 children who had laparotomy after primary therapy because of suspected intra-abdominal relapse had this diagnosis confirmed by the procedure: all had splenic involvement.

None of the 19 liver and iliac-crest biopsy specimens in the two groups were positive. There were no serious complications after any of the 19 laparotomies, and in particular no patient has developed pneumococcal septicaemia, a recognized complication of splenectomy in childhood (Chilcoate *et al.*, 1975).

Since the beginning of 1976, it has been our practice to give prophylactic oral penicillin to all our splenectomized patients under the age of 15, and this may account for their freedom from late infective complications.

TABLE III.—*Sites of Relapse in Patients Treated with Extended-field (Mantle) Radiotherapy after Adequate Staging*

| Patient | Age | Sex | Histology | Clinical stage | Staging laparotomy (findings) | Site of relapse |
|---|---|---|---|---|---|---|
| T.Ha. | 15 | F | NS | IIB (ad)* | Yes (— ve) | Local recurrence (hilum and lung) |
| G.K. | 15 | M | NS | IIIB | Yes (— ve) | Local recurrence |
| T.H. | 15 | F | NS | IIA (ad) | No | Spleen |
| A.S. | 9 | M | NS | IIA (ad) | No | Spleen |
| C.Y. | 15 | M | LP | IIA (ad) | No | Marginal recurrence (right submandibular node) |
| E.F. | 11 | M | LP | IIB (ad) | No | Spleen and para-aortic nodes |
| C.L. | 14 | F | NS | IIA (ad) | No | Spleen and lung |

* ad = above diaphragm.

TABLE IV.—*Findings at Staging Laparotomy in Children with Hodgkin's Disease*

| Patient | Age | Sex | Histology | Clinical sites of involvement (clinical stage) | Laparotomy finding | Spleen | Nodes | Porta hepatis node | Liver | Marrow |
|---|---|---|---|---|---|---|---|---|---|---|
| M.J. | 8 | M | MC | L. neck, spleen (IIIA) | + | + | + | + | − | − |
| P.K. | 13 | M | MC | R. neck, para-aortic nodes (IIIA) | + | + | + | + | − | − |
| T.W. | 15 | M | NS | L. neck, R. groin (IIIB) | + | + | + | − | − | − |
| C.W. | 12 | F | NS | R. neck, R. and L. axillae, spleen (IIIA) | + | − | − | + | − | − |
| A.A. | 10 | M | NS | R. neck and mediastinum, spleen (IIIA) | + | + | + | − | − | − |
| W.B. | 13 | F | NS | R. and L. neck, nasopharynx (IIA) | − | − | − | − | − | − |
| T.H. | 15 | F | NS | L. neck, mediastinum, pleural effusion (IIE B) | − | − | − | − | − | − |
| G.K. | 15 | M | NS | R. and L. neck, mediastinum, para-aortic nodes (IIIB) | − | − | − | − | − | − |
| T.W. | 15 | M | LP | Left neck (IA) | − | − | − | − | − | − |
| I.B. | 12 | F | MC | Left neck, mediastinum (IIA) | − | − | − | − | − | − |
| R.G. | 12 | M | NS | R. neck (IA) | − | − | − | − | − | − |
| C.C. | 11 | M | NS | L. neck (IA) | − | − | − | − | − | − |
| C.S. | 13 | F | NS | L. neck, mediastinum (IIA) | − | − | − | − | − | − |
| Total | 13 | | | | 5 | 4 | 4 | 3 | 0 | 0 |

*Late complications after primary therapy*

Two children developed hypothyroidism one and 3 years after mantle irradiation. There were no serious growth disturbances in the group, except that one boy showed slight maldevelopment of the thoracic cage with depressed respiratory function tests, 7 years after mantle irradiation.

Two second malignancies occurred: at the age of 19, one girl developed the bone-marrow and peripheral-blood appearances of chronic lymphocytic leukaemia, 3 years after therapy which included both mantle irradiation and 10 courses of MVPP; a second girl, aged 20 developed carcinoma of the uterine cervix 8 years after inverted "Y" radiotherapy and 4 years after MVPP chemotherapy. It is of interest that the cervical cancer was preceded by multiple vulval and vaginal condylomas.

### DISCUSSION

Hodgkin's disease in children behaves in a similar way to the disease in adults. These are geographical differences in the incidence of childhood Hodgkin's disease (McMahon, 1966) but in western countries the disease is uncommon in children, particularly in the very young, and increases with age (Fraumeni and Li, 1969; Jenkin *et al.*, 1967; Young *et al.*, 1973). The higher incidence in males than in females in our study confirms the experience of others (Fraumeni and Li, 1969; Schnitzer *et al.*, 1973; Strum and Rappaport, 1970; Tan *et al.*, 1975; Young *et al.*, 1973) and, at least in the very youngest age groups, is much more marked than the slightly increased male incidence reported for adults (McMahon, 1966).

In this study, more than 70% of children treated with chemotherapy or radiotherapy, based in most instances on clinical staging, are disease free and this, together with the predicted overall median survival of 10 years, reflects the benefits of modern therapy, as others have also demonstrated (Jenkin *et al.*, 1975; Tan *et al.*, 1975). It is clear that therapy should be designed to minimize complications and long-term sequelae, as much as to improve techniques for disease eradication. However, low-dose involved-field radiotherapy in patients who have been inadequately staged results in a 3-year complete remission rate of only 13%, and is inadequate for controlling disease. Extended-field radiotherapy, including adjacent lymph-node groups, after adequate staging with lymphography, gave a much better disease-free survival with a 3-year rate of 72%. Others have reported similar results (Jenkin *et al.*, 1975; Young *et al.*, 1973), and it is important to consider, with these results in mind, whether extended-field radiotherapy should be the treatment of choice for localized disease in children, as it is now for adults. In this context the potential hazards of bone-growth impairment and organ maldevelopment need to be considered. Probert, Parker and Kaplan (1974) reported a retardation of spinal growth in 16/22 children receiving complete spinal irradiation, but it is important to point out that the doses employed were considerably higher than those habitually used at this centre. Young *et al.* (1973) noted retardation in vertebral body growth after extended-field radiotherapy, particularly in younger children; and one child in our series has developed thoracic deformity and impaired lung function after mantle irradiation, but again, after receiving a dose of more than 4000 rad to a generous field. In children as in adults, hypothyroidism may result from irradiation of the thyroid. Gonadal irradiation may result in infertility, but does not appear to interfere with the other normal changes occurring at puberty.

It is important, therefore, to question the routine use of this extended-field approach, particularly in early childhood, and clearly pertinent to this is the performance of chemotherapy as a method of primary treatment of Hodgkin's disease in children. It is also important to identify children who may respond satisfactorily to local field irradiation for Stage I disease. In our study, 3 children with Stage IA upper-cervical-node involvement had elective local radiotherapy limited to both sides of the neck, and remain disease-free. This experience is obviously too small to allow us to draw conclusions, but Fuller *et al.* (1973) previously reported that local radiotherapy in children adequately staged by lymphography and laparotomy may be as effective as extended-field radiotherapy, with less risk of growth complications. The importance of adequate staging where such therapy is to be contemplated cannot be over-emphasized: lymphography is an essential staging procedure and there is a good argument for laparotomy and splenectomy prior to irradiation, at least in children of 11 years and older, since the radiotherapy results obtained in adults staged by laparotomy have shown a considerable improvement over those obtained in clinically staged patients (Peckham *et al.*, 1975).

Combination chemotherapy achieved a remission rate comparable to that in adults. So far, the results in the chemotherapy group are comparable with those observed in the radiotherapy group (Fig. 6). This finding must be qualified by the small number of patients in the chemotherapy group, and by the fact that 7/10 children treated primarily by chemotherapy are still less than 2 years from the start of treatment. Nevertheless, our data so far support those of Ziegler *et al.* (1972) and Olweny *et al.* (1974) who gave combination chemotherapy to Ugandan children with early-stage disease, albeit of different histological distribution to our series, and achieved results comparable with other series where treatment was by conventional extended-field radiotherapy. Combination chemotherapy has its own disadvantages: sterility in males, at least for a time after treatment, is almost inevitable (De Vita *et al.*, 1973) although the extent to which this will remain a long-term problem in male children is so far unknown. The induction of second tumours is a risk which remains to be assessed. The major attraction of chemotherapy, particularly in young children, is the avoidance of the sequelae of irradiation in growing bone, since serious problems of growth disturbance do not appear to occur with chemotherapy (Young *et al.*, 1973) and certainly local malformations after therapy are not a problem. Chemotherapy, if employed alone, further obviates the need for a staging laparotomy with splenectomy. Finally, even if relapse does occur after chemotherapy, radiotherapy may well remain a curative treatment in many instances, and perhaps at a later stage in the child's development, when growth problems are less likely.

Laparotomy with splenectomy as a staging procedure in childhood Hodgkin's disease presents a difficult problem. Its rationale, as in adults, is to diagnose or exclude occult intra-abdominal disease, especially in the spleen, and so to plan more confidently an irradiation field that should be effective without including more uninvolved normal tissue than is necessary. On this basis, the procedure has a particular attraction in children, in whom the importance of limiting the irradiation field and hence the risk of long-term sequelae has been emphasized. However, the operation is not a minor one, and while our group of children has escaped serious post-operative complications, severe infections and deaths have been recorded in children after the procedure (Jenkin *et al.*, 1975; Hays *et al.*, 1972; Rosenberg, 1971).

More recently Chilcoate *et al* (1975) have documented 20 episodes of septicaemia and/or meningitis in 200 children, following diagnostic laparotomy and splenectomy. Eleven of these children died. They emphasize the frequency of penicillin-sensitive organisms, the most frequent being *D. pneumoniae*, and we would strongly support their recommendation that splenectomized children should receive prophylactic penicillin.

Five of the 13 children undergoing laparotomy in our group had positive findings and, although in none of these was staging or treatment changed, no child in the group had an intra-abdominal relapse after laparotomy, whereas 4 children relapsed in spleen or abdominal nodes after complete clinical staging but without laparotomy; conceivably, occult disease might have been diagnosed and eradicated in some of these at diagnosis, had they undergone the procedure. A more clearly established role for chemotherapy in early stage disease would, of course, bring with it the benefit of avoiding the need for the operation; but at present we feel that in children of 11 years and older, the advantages of laparotomy and splenectomy outweigh the possible risks, and that the procedure should be carried out where radiotherapy which includes possible intra-abdominal sites of disease is contemplated. This policy may need to be modified in the light of future experience with chemotherapy.

It is clear that the management of children's Hodgkin's disease needs very considerable individualization, with disease site, histology and particularly age of the patient being taken into account. Patients in later childhood, for example, can be managed essentially as adults, whilst in the younger child, avoidance of surgical staging procedure and extended-field radiotherapy may be of paramount importance.

We can, therefore, summarize our present philosophy of treatment as follows:

In children of 10 years or less, where laparotomy would be a hazard, lymphography is performed, and they are then treated with chemotherapy when there is a positive lymphograph or where histology (MC or LD) indicates that there is a very substantial risk of abdominal involvement. In children with localized disease and prognostically favourable histology (LP or NS) local irradiation to the tumour-bearing lymph node area is given first. Children with Stage IV disease are treated with chemotherapy.

In children of 11 years and over the patients are pathologically staged by laparotomy and splenectomy (unless there is obvious Stage IV disease). Children with pathological Stages I or II disease are treated with extended-field radiotherapy unless there are more than 3 nodal areas involved, particularly when infra-clavicular node involvement or a large mediastinal mass are present, since these features are known to be associated with a very low cure rate for radiotherapy alone (Peckham *et al.*, 1975). Here chemotherapy is given first, to achieve good tumour regression before irradiation. In Stage IIIA (with a negative spleen) treatment will depend upon the age of the child. It is our policy to treat children of 13 or over with total nodal irradiation as in adults. In younger children we prefer to use chemotherapy, in an attempt to minimize damage to the growing spine. When the spleen is involved, and in Stage IIIB disease, chemotherapy, which may be followed by irradiation to sites of bulky disease, is given. In Stage IV, chemotherapy alone is given, as it is in adults. All splenectomized children receive oral prophylactic penicillin until the age of 15 years.

### REFERENCES

CARBONE, P. P., KAPLAN, H. S., MUSSHOFF, K., SMITHERS, D. W. & TUBIANA, M. (1971) Report of the Committee on Hodgkin's Disease Staging Classification. *Cancer Res.*, **31**, 1860.

CHILCOATE, R. R. & BAEHNER, R. L. (1975) The Incidence of Overwhelming Infection in Children Staged for Hodgkin's Disease. *Proc. Am. Ass. Cancer Res.*, **16**, 224.

DE VITA, V. T., SERPICK, A. A. & CARBONE, P. P. (1970) Combination Chemotherapy in the Treatment of Advanced Hodgkin's Disease. *Ann. intern. Med.*, **73**, 881.

DE VITA, V. T., ARSENEAU, J. C., SHERINS, R. J., CANELLOS, G. P. & YOUNG, R. C. (1973) Intensive Chemotherapy for Hodgkin's Disease: Long Term Complications. *Natn. Cancer Inst. Monog.*, **36**, 447.

FRAUMENI, J. F. & LI, F. P. (1969) Hodgkin's Disease in Childhood: An Epidemiologic Study. *J. natn. Cancer Inst.*, **42**, 681.

FULLER, L. M., SULLIVAN, M. P. & BUTLER, J. J. (1973) Results of Regional Radiotherapy in Localized Hodgkin's Disease in Children. *Cancer, N.Y.*, **32**, 640.

GAZET, J.-C. (1973) Laparotomy and Splenectomy. In *Hodgkin's Disease*. Ed. D. W. Smithers. Edinburgh: Churchill Livingstone. p. 190.

HAYS, D. M., HITTLE, R. E., ISAACS, H., JR. & KARON, M. R. (1972) Laparotomy for the Staging of Hodgkin's Disease in Children. *J. Pediat. Surg.*, **7**, 517.

JENKIN, R. D. T., BROWN, T. C., PETERS, M. V. & SUNLEY, M. J. (1975) Hodgkin's Disease in Children. *Cancer, N.Y.*, **35**, 979.

JENKIN, R. D. T., PETERS, M. V. & DARTE, J. M. M. (1967) Hodgkin's Disease in Children. *Am. J. Roentg.*, **100**, 222.

LUKES, R. J. & BUTLER, J. J. (1966) The Pathology and Nomenclature of Hodgkin's Disease. *Cancer Res.*, **26**, 1063.

MCELWAIN, T. J., WRIGLEY, P. F. M., HUNTER, A., CROWTHER, D., MALPAS, J. S., PECKHAM, M. J., SMITHERS, D. W. & FAIRLEY, G. H. (1973) Combination Chemotherapy in Advanced and Recurrent Hodgkin's Disease. *Natl. Cancer Inst. Monogr.*, **36**, 395.

MCMAHON, B. (1966) Epidemiology of Hodgkin's Disease. *Cancer Res.*, **26**, 1189.

OLWENY, C. L. M., MBIDDE, E. K., NKWOCHA, J., MAGRATH, I. T. & ZIEGLER, J. L. (1974) Chemotherapy of Hodgkin's Disease. *Lancet*, ii, 1397.

PECKHAM, M. J. (1973) The Radiotherapy of Hodgkin's Disease. *Br. J. Hosp. Med.*, **9**, 457.

PECKHAM, M. J., FORD, H. T., MCELWAIN, T. J., HARMER, C. L., ATKINSON, K. & AUSTIN, D. E. (1975) The Results of Radiotherapy for Hodgkin's Disease. *Br. J. Cancer*, **32**, 391.

PROBERT, J. C., PARKER, B. R. & KAPLAN, H. S. (1974) Growth Retardation in Children after Megavoltage Irradiation of the Spine. *Radiology*, **111**, 766.

ROSENBERG, S. A. (1971) A Critique of the Value of Laparotomy and Splenectomy in the Evaluation of Patients with Hodgkin's Disease. *Cancer Res.*, **31**, 1737.

SCHNITZER, B., NISHIYAMA, R. H., HEIDELBERGER, K. P. & WEAVER, D. K. (1973) Hodgkin's Disease in Children. *Cancer, N.Y.*, **31**, 560.

STRUM, S. B. & RAPPAPORT, H. (1970) Hodgkin's Disease in the First Decade of Life. *Pediatrics*, **46**, 748.

TAN, C., D'ANGIO, G. J., EXELBY, P. R., LIEBERMAN, P. H., WATSON, R. C., CHAM, W. C. & MURPHY, M. L. (1975) The Changing Management of Childhood Hodgkin's Disease. *Cancer, N.Y.*, **35**, 808.

YOUNG, R. C., DE VITA, V. T. & JOHNSON, R. E. (1973) Hodgkin's Disease in Childhood. *Blood*, **42**, 163.

ZIEGLER, J. L., BLUMING, A. Z., FASS, L., MAGRATH, I. T. & TEMPLETON, A. C. (1972) Chemotherapy of Childhood Hodgkin's Disease in Uganda. *Lancet*, ii, 679.